# Examining the association between the *FTO* gene and neuroticism reveals indirect effects on subjective well-being and problematic alcohol use

**DOI:** 10.1038/s41598-024-68578-2

**Published:** 2024-07-30

**Authors:** Wenjie Cai, Yvonne Forsell, Catharina Lavebratt, Philippe A. Melas

**Affiliations:** 1 Department of Molecular Medicine and Surgery, Karolinska Institutet, 17176 Stockholm, Sweden; 2 Center for Molecular Medicine, L8:00, Karolinska University Hospital, 17176 Stockholm, Sweden; 3 Department of Global Public Health, Karolinska Institutet, 17177 Stockholm, Sweden; 4 Center for Psychiatry Research, Department of Clinical Neuroscience, Karolinska Institutet and Stockholm Health Care Services, 11364 Stockholm, Sweden

**Keywords:** Fat mass and obesity-associated gene, Alpha-ketoglutarate-dependent dioxygenase FTO, *ALKBH9*, Big five personality traits, Alcohol use disorder, AUD, Alcoholism, Genetics, Behavioural genetics, Genetic association study, Psychiatric disorders, Addiction

## Abstract

Associations between the fat mass and obesity-associated (*FTO*) gene and obesity are well-established. However, recent studies have linked *FTO* to addiction phenotypes and dopaminergic signaling, thus suggesting broader psychiatric implications. We explored this assumption by conducting a phenome-wide association study across 4756 genome-wide association studies, identifying 23–26 psychiatric traits associated with *FTO* at the multiple-corrected significance level. These traits clustered into four categories: substance use, chronotype/sleep, well-being, and neuroticism. To validate these findings, we analyzed a functionally suggestive *FTO* variant (rs1421085) in a separate cohort, examining its impact on (i) alcohol use based on the Alcohol Use Disorders Identification Test (AUDIT), (ii) subjective well-being based on the WHO (Ten) Well-Being Index, and (iii) neuroticism based on Schafer’s Five Factor Model or the Karolinska Scales of Personality. Our results confirmed a direct association between rs1421085 and neuroticism that was independent of age, sex, alcohol use, body mass index (BMI), and childhood adversities. Interestingly, while no direct association with alcohol intake was observed, both cross-sectional and lagged longitudinal mediation analyses uncovered indirect relationships between rs1421085 and problematic alcohol use (AUDIT-P), with increased neuroticism acting as the intermediary. Mediation analyses also supported an indirect effect of rs1421085 on lower well-being through the pathways of increased neuroticism and BMI. Our study is the first to validate a direct association between *FTO* and neuroticism. However, additional studies are warranted to affirm the causal pathways linking *FTO* to well-being and alcohol use through neuroticism.

## Introduction

The gene encoding the alpha-ketoglutarate-dependent dioxygenase (*ALKBH9)*, popularly known as the fat mass and obesity-associated gene (*FTO*), is widely recognized for its role as a genetic factor predisposing to obesity^1,2^. More recently, however, genome-wide association studies (GWASs) have reliably associated *FTO* with an elevated risk of various alcohol use traits, independent of body mass index (BMI) effects^3–6^. Additionally, a GWAS examining multiple substance use disorders suggested that *FTO* may represent a broader risk factor for addiction^7^. More specifically, single nucleotide polymorphisms (SNPs) within the first intronic region of *FTO* have been associated with alcohol use disorder (rs1421085)^3^, alcohol consumption (rs62033408)^3^, and problematic alcohol use (rs9937709, rs1421085)^4,8^. Multitrait analysis of GWAS (MTAG) identified associations between *FTO* (rs7188250) and both maximum habitual alcohol intake and problematic alcohol use^5^. The largest GWAS of drinking phenotypes so far, encompassing nearly 3.4 million individuals, found associations between *FTO* variants and weekly alcohol consumption (rs1421085, rs11642015, rs62048402, rs1558902)^6^. Furthermore, a gene-based analysis within a multivariate GWAS assessing a general addiction risk factor, found that *FTO* was the most significant gene involved (fine-mapped SNP: rs1477196)^7^.

While much of the *FTO* research has concentrated on obesity, its potential involvement in neuropsychiatric disorders is gaining traction, supported by both preclinical and translational studies^9^. *FTO* encodes an m6A RNA demethylase, highly enriched in neurons, and plays a role in neurotransmission and memory formation, including adult neurogenesis^10–16^. Notably, deactivation of the *Fto* gene in animal models disrupts D2 and D3 dopamine receptor-dependent control of neuronal activity and behavioral responses^11^. Given the dopaminergic system's extensive involvement in various psychiatric disorders^17^, and the known comorbidity of substance use disorders with these conditions^18^, we postulated that *FTO* may influence a broader range of psychiatric traits beyond the addiction phenotypes already identified. To explore this, we conducted a phenome-wide association study (PheWAS) and followed up with genotyping a functionally relevant SNP in a separate cohort. Our research contributes to understanding the broader role of *FTO* in psychiatric traits and aligns with the importance of considering personality traits such as neuroticism in developing intervention strategies.

## Methods

### Phenome-wide association study (PheWAS)

To investigate the broader implications of the *FTO* gene, we conducted a PheWAS utilizing the Atlas of GWAS Summary Statistics (GWAS Atlas, https://atlas.ctglab.nl/, last accessed November 9, 2023)^19^. This analysis encompassed 4,756 GWAS datasets, covering 3302 unique phenotypic traits. A PheWAS plot was generated to visualize associations for 791 traits meeting the nominal p-value cutoff of 0.05. Bonferroni correction was first applied across the 791 traits with *p* < 0.05, as provided by the PheWAS function of the GWAS Atlas, setting the significance threshold at a *p*-value of 6.32e-5 (0.05/791). We also performed a Bonferroni correction for all 3302 phenotypes tested in the PheWAS to control for Type I errors more stringently, which set the significance threshold at a p-value of 1.514e-5 (0.05/3302).

### Replication cohort: cross-sectional and longitudinal data

Our replication phase leveraged data from PART, a longitudinal cohort study from Stockholm County, Sweden, which relies on self-administered questionnaires (validated by interviews with psychiatrists) to explore risk and protective factors for mental health^20^. For cross-sectional analyses, and unless otherwise indicated, we extracted and analyzed data on demographics (age and sex), anthropometrics (BMI) and psychiatric-related traits (alcohol use, well-being, and neuroticism) from the third wave of PART (PART wave III; 2010–2011)^21^, which was the only wave to comprehensively assess neuroticism using the Schafer’s Five Factor Model (FFM) personality scale (see below). For longitudinal mediation analyses, data on BMI, alcohol use, well-being, and a subset of neuroticism-related items from the Karolinska Scales of Personality (KSP; see below), were also extracted from the second wave of PART (PART wave II; 2001–2003)^22^. The PART study cohort is predominantly Swedish, with only 11% of participants being of non-Swedish origin, and within this subset the overwhelming majority are of Nordic descent, primarily Finnish^20^. At the time of sample collection, the Swedish population was characterized by minimal internal genetic differentiation, particularly in the southern and central regions, where our participants are predominantly located^23,24^. Moreover, previous analyses focusing solely on participants of Swedish origin yielded statistically significant results consistent with those derived from the entire cohort^25^. Thus, although a specific test for population stratification was not conducted, this inherent genetic homogeneity suggests that the risk of population stratification confounding our findings is substantially minimized. Ethical approval for PART was obtained from the Karolinska Institutet's review board (nr. 96-260 and 97-313 for questionnaire data, nr. 2004-528/3 for DNA data, and nr. 2009/880-31 for PART wave III), in line with the World Medical Association's (WMA) Helsinki Declaration. All participants provided informed consent, and all described methods were carried out in accordance with relevant guidelines and regulations.

### Alcohol use measurement

Alcohol use was assessed in the PART study using the 10-item Alcohol Use Disorders Identification Test (AUDIT), which examines drinking habits and associated problems over the past 12 months, as previously described^26,27^. The analysis included the full AUDIT scale (items 1–10), the AUDIT-C subscale focusing on consumption (items 1–3), and the AUDIT-P subscale targeting problematic usage (items 4–10).

### Subjective well-being measurement

Subjective well-being in the PART study was measured using the WHO (Ten) Well-Being Index, a derivative of the WHO (Bradley) Subjective Well-Being Inventory Index, as previously described^28–30^. This 10-item scale ranges from 0 (never) to 3 (always), reflecting the individual’s state over the previous week. The responses to the 10 items were summed to create a total well-being score, with higher scores indicating greater well-being.

### Neuroticism measurements

Neuroticism was comprehensively assessed in PART wave III, as part of the Swedish-translated Schafer’s Five Factor Model (FFM) personality scale^31–34^, which evaluates the ‘Big Five’ personality traits, i.e., extraversion, agreeableness, conscientiousness, openness, and neuroticism. Although the FFM scale was not incorporated in the previous PART waves, PART wave II included 14 neuroticism-related items from the Karolinska Scales of Personality (KSP). Specifically, the KSP inventory features items related to psychic anxiety, somatic anxiety, muscular tension, psychasthenia (lack of energy), inhibition of aggression (lack of assertiveness), guilt, suspicion, indirect aggression and irritability, all of which correlate positively with neuroticism in the Eysenck Personality Questionnaire-Revised (EPR-R) scale^35^. FFM- and KSP-derived neuroticism scores were standardized into z-scores, and there was a significant positive correlation between them (Pearson’s r = 0.571, *p* < 0.001).

### Childhood adversities

Information on childhood adversities was obtained from the first wave of PART (PART wave I; 1998–2000)^36^. These adversities, experienced before the age of 18, included severe family conflicts, significant financial hardships in the family, parental divorce, or the loss of a parent, and were coded categorically as either the absence of adversities or the presence of at least one. Recognizing the established association of childhood adversities with BMI^37,38^, substance use^39,40^, subjective well-being^41,42^, and neuroticism^43,44^, we accounted for them as covariates in our regression analyses (see below).

### Genotyping

A sub-cohort of the PART study provided DNA samples via self-administered saliva collection kits (Oragene DNA sample collection kit; DNA Genotek Inc., Stittsville, ON, Canada), as previously described^45,46^. The rs1421085 SNP in *FTO* was genotyped using the TaqMan Universal Master Mix and a TaqMan SNP genotyping assay on an ABI 7900 HT instrument (Thermo Fisher Scientific, Waltham, MA, USA). This SNP was chosen based on its functional significance according to the RegulomeDB, the Brain eQTL Almanac (Braineac) and the GTEx database, as detailed in the Results section.

### Statistical analyses

Bivariate correlations between continuous variables were assessed using Pearson's correlation coefficient. Linear regressions were used to examine the association of rs1421085 with neuroticism, well-being, BMI, and AUDIT scores in the PART cohort. The genotypic effects were modeled dominantly, with the T allele posited as the effect allele based on previous GWAS findings^3^ and functional annotations from databases such as RegulomeDB and Braineac. In this model, the TT and CT genotype groups were collapsed into a single group to represent the presence of the T allele. The choice of a dominant model is supported by several factors, including a biological plausibility of regulatory variants exerting effects in a single and a double copy, consistency with our hypothesis-driven approach to validate specific associations observed in prior studies, and increased statistical power in detecting associations with moderate effect sizes. Multicollinearity diagnostics were conducted using variance inflation factor (VIF) and tolerance metrics. Interaction terms were included in the regression models to assess the potential moderating effects of neuroticism and well-being on the relationship between the *FTO* genotype and the outcomes of interest. Specifically, we tested the following interaction terms: (i) Genotype (2 levels: T allele carriers vs. CC homozygotes) * Neuroticism (quantitative phenotype). (ii) Genotype (2 levels: T allele carriers vs. CC homozygotes) * Well-being (quantitative phenotype). Statistical analyses were performed using IBM SPSS Statistics v.27 (IBM Corp, Armonk, NY, USA), with alpha set at 0.05. Given the candidate gene approach for the PART replication cohort analyses, we did not apply multiple testing corrections, as the analyses were hypothesis-driven rather than exploratory. Cross-sectional and lagged longitudinal mediation analyses were conducted using Model 4 of the PROCESS macro for SPSS^47^, which performs a simple mediation analysis. In this model, the independent variable (X) affects the dependent variable (Y) through a mediator (M). The analysis estimates both the direct effect of X on Y and the indirect effect of X on Y through M. The indirect effect is calculated using bias-corrected bootstrap confidence intervals, with 5000 resamples to ensure robust estimates.

## Results

### PheWAS analysis reveals FTO gene associations across psychiatric traits

The phenome-wide association study (PheWAS) of the *FTO* gene considered 4,756 GWAS datasets encompassing a total of 3,302 phenotypic traits and yielded nominally significant associations with 791 traits across 27 domains (Fig. S1). Out of these, 242 and 224 traits across 17 domains met the criteria for multiple-testing corrected significance at the 6.32e-5 and the 1.514e-5 threshold levels, respectively (File S1). The most pronounced PheWAS signals for *FTO* resided within the metabolic domain and included body mass index (BMI), obesity status, waist-hip ratio, and various bioelectrical impedance measures (Fig. S1 and File S1). Within the psychiatric domain, 26 and 23 traits survived multiple testing at the 6.32e-5 and 1.514e-5 thresholds, respectively, and were grouped into four categories (Table 1). More specifically, as shown in Table 1, the substance use category was highlighted by a leading association with alcohol consumption. The three other categories included traits related to chronotype/sleep, well-being, and neuroticism. There was also one trait related to a symptom of depression, i.e., recent poor appetite or overeating, which we attributed to the metabolic domain given its connection to caloric intake. In summary, the PheWAS findings underscore the diverse impact of the *FTO* gene, revealing its significant associations with a broad spectrum of psychiatric traits, most notably in substance use, chronotype and sleep, subjective well-being, and neuroticism, thus extending its known influence beyond metabolic traits.

**Table 1:** PheWAS of the *FTO* gene reveals associations with psychiatric traits related to substance use, chronotype/sleep, well-being, and neuroticism.

Trait^‡^	P-value^a^	N^b^	Category^c^
Average weekly beer plus cider intake	3.12E-21	274,556	Substance Use
Chronotype	7.52E-21	449,732	Chronotype/sleep
Morningness	3.94E-18	345,552	Chronotype/sleep
Morning/evening person (chronotype)	9.08E-18	345,148	Chronotype/sleep
Morning person (binary)	5.11E-17	403,195	Chronotype/sleep
Sleep duration	6.17E-14	384,225	Chronotype/sleep
Sleep duration	6.97E-14	446,118	Chronotype/sleep
Sleep duration	1.45E-13	384,317	Chronotype/sleep
Alcohol intake frequency	5.15E-13	386,082	Substance Use
Ease of getting up in the morning	2.78E-10	385,949	Chronotype/sleep
Getting up in morning	4.15E-10	385,494	Chronotype/sleep
Snoring	1.71E-09	359,916	Chronotype/sleep
Snoring	2.69E-09	359,498	Chronotype/sleep
Depression—Recent poor appetite or overeating	1.07E-08	126,639	Metabolic domain
Long sleep	8.62E-08	339,926	Chronotype/sleep
Ever smoker	2.61E-07	518,633	Substance Use
Drinks per day	4.26E-07	537,349	Substance Use
Alcohol dependence	5.15E-07	2322	Substance Use
Happiness and subjective well-being—General happiness with own health	5.85E-07	126,477	Well-being
Drinks per week	9.08E-07	414,343	Substance Use
10 mg response to amphetamine	3.8017E-06	381	Substance Use
Sleep duration (mean)	8.7287E-06	85,449	Chronotype/sleep
Irritability	9.2669E-06	369,232	Neuroticism
Past tobacco smoking^**‡‡**^	2.1418E-05	355,594	Substance Use
Ever smoked^**‡‡**^	3.5718E-05	385,013	Substance Use
Neuroticism^**‡‡**^	3.958E-05	390,278	Neuroticism

^‡^Traits from the psychiatric domain that passed the Bonferroni corrected P-value of 6.32e-5.

^‡‡^Traits from the psychiatric domain that did not pass the more stringent Bonferroni corrected P-value of 1.514e-5.

^a^*P*-values are ranked from lowest to highest.

^b^Total sample size in respective GWAS.

^c^Traits were classified into four psychiatric categories, i.e., substance use, chronotype/sleep, well-being, and neuroticism. One depression-related symptom (i.e., recent poor appetite or overeating) was attributed to the metabolic domain given its relationship to caloric intake.

### rs1421085: A putative regulatory variant within the FTO gene

Next, we aimed at replicating the associations of *FTO* with psychiatric traits by identifying and genotyping proxy SNP(s) with putative functionality. Among the eight SNPs associated with substance use phenotypes in GWAS studies (i.e., rs1421085, rs62033408, rs9937709, rs7188250, rs11642015, rs62048402, rs1558902, rs1477196)^3–8^, rs1421085 had the highest possible functional score on RegulomeDB (probability score ≈ 1, ranking score: 1a; Table S1). Complementing this finding, the Brain eQTL Almanac (Braineac) provided supportive evidence for rs1421085 influencing *FTO* gene expression in neural tissues. Specifically, the T-allele, showed a nominal increase in *FTO* expression within the occipital cortex and cerebellum (Fig. S2), aligning with previous GWAS findings that identified the T allele as influential^3^. The GTEx portal's data also provided evidence for a regulatory role of rs1421085 in *FTO* expression in muscle, small intestine, pancreas, skin, and thyroid tissues (Fig. S3). It is also worth noting the significant correlation between rs1421085 and the other substance use-related SNPs, as established through linkage disequilibrium (LD) statistics. The LD measures generated by the LDpair Tool for the European population demonstrated a high degree of correlation with rs62033408 (D’: 1.0, R2: 0.918, *p* < 0.0001), rs9937709 (D’: 0.950, R2: 0.874, *p* < 0.0001), rs7188250 (D’: 0.937, R2: 0.840, *p* < 0.0001), rs11642015, rs62048402, and rs1558902 (D’: 1.0, R2: 1.0, *p* < 0.0001 for all). However, it was less correlated with rs1477196 (D’: 1.0, R2: 0.41, *p* < 0.0001). Collectively, these data suggest that rs1421085 is an *FTO* variant with likely functional consequences and a suitable proxy in subsequent genotyping studies.

### Demographic and genotypic characteristics of the replication cohort

For our replication study, we leveraged data from the PART study which includes longitudinal measures on BMI, alcohol use, subjective well-being, and neuroticism (data on chronotype/sleep were not available). We genotyped rs1421085 in a cohort of 2194 PART participants with females representing 60.3% of the total sample. The average age of genotyped participants was 56.6 years, with a standard deviation (SD) of 11.9 years, and a range from 31 to 77 years. Table S2 provides detailed descriptive statistics for the quantitative phenotypes, including the range, mean, and standard deviation for AUDIT scores, well-being scores, neuroticism measures, and BMI. The number and percentage of participants with or without childhood adversities are provided in Table S3. The genotype distribution for rs1421085 was consistent with the Hardy–Weinberg equilibrium (χ^2^ = 0.0097, *p* = 0.92). Moreover, the observed genotype frequencies [TT: 810 individuals (36.9%), CT: 1048 individuals (47.8%), CC: 336 individuals (15.3%)] aligned with the allele frequencies reported in the broader population by dbGaP (T allele frequency = 0.608, C allele frequency = 0.391, based on a sample size of 299,532 individuals; source: Alpha Allele Frequency release version: 20201027095038). Collectively, the genotypic data reinforce the representativeness of the replication cohort for investigating the genetic influences of the *FTO* variant rs1421085 on psychiatric traits.

### Validation of FTO’s associations with neuroticism and subjective well-being

In the replication cohort from the PART study, we started by exploring the association between the *FTO* gene and selected psychiatric-related traits, including alcohol use, neuroticism, and subjective well-being. We conducted linear regression analyses using the rs1421085 genotype under a dominant model of genetic effect, where the T allele was considered the effect allele according to previous GWAS findings (see also Statistical Analyses in Methods)^3^. The analysis revealed that carriers of the T allele demonstrated significantly higher neuroticism scores; a finding that was consistent in both the unadjusted model (Table 2; model a, B: 0.12, *p* = 0.04) and the model accounting for age, sex, BMI, alcohol use, and childhood adversities (Table 2; model b, B: 0.16, *p* = 0.007). Upon examining subjective well-being, a significant inverse association with the rs1421085 genotype emerged in the model adjusted for age, sex, BMI, alcohol use, and childhood adversities, where T-allele carriers reported lower well-being levels (Table 3; model b, B: −0.90, *p* = 0.01). Notably, this association did not reach significance in the unadjusted analysis (Table 3; model a, B: −0.6, *p* = 0.1). In contrast, alcohol use, as measured by the AUDIT scales, did not show a significant association with rs1421085 in any model (Table S4). As a confirmatory analysis, we also examined the association between rs1421085 and BMI, and found it to be significant in both unadjusted and adjusted models, with T-allele carriers having lower BMI (Table S5). Table S6 provides the means and standard errors for neuroticism, well-being, AUDIT, and BMI by rs1421085 genotype. In summary, this validation phase extends the known association between *FTO* and BMI, to also include associations with higher levels of neuroticism and reduced subjective well-being, affirming the gene’s significant role in these psychiatric-related dimensions.

**Table 2:** Linear regression of neuroticism on the *FTO* rs1421085 genotype^‡^

N	B (95% CI)^a^	*P*	N	B (95% CI)^b^	*P*	N	B (95% CI)^c^	*P*
2,150	0.12 (0.002, 0.24)	0.04	1890	0.16 (0.04, 0.28)	0.007	1819	0.08 (−0.02, 0.18)	0.11

B: Unstandardized beta coefficient, CI: Confidence Interval, P: *p*-value.

^‡^T-allele carriers (CT or TT; coded 1) compared to CC homozygotes (CC; coded 0).

^a^Crude regression.

^b^Adjusted for age, sex, BMI, AUDIT-10, childhood adversities.

^c^Adjusted for age, sex, BMI, AUDIT-10, childhood adversities, well-being.

**Table 3:** Linear regression of subjective well-being on the *FTO* rs1421085 genotype^‡^

N	B (95% CI)^a^	*P*	N	B (95% CI)^b^	*P*	N	B (95% CI)^c^	*P*
2094	−0.60 (−1.33, 0.12)	0.10	1847	−0.90 (−1.64, −0.16)	0.01	1819	−0.29 (−0.90, 0.30)	0.33

B: Unstandardized beta coefficient, CI: Confidence Interval, P: *p*-value.

^‡^T-allele carriers (CT or TT; coded 1) compared to CC homozygotes (CC; coded 0).

^a^Crude regression.

^b^Adjusted for age, sex, BMI, AUDIT-10, childhood adversities.

^c^Adjusted for age, sex, BMI, AUDIT-10, childhood adversities, neuroticism.

### Exploring the indirect role of neuroticism in FTO’s association with well-being

Our replication efforts confirmed the connection between the *FTO* gene and both neuroticism and subjective well-being. As expected, there was a significant negative correlation between neuroticism and well-being within our cohort (Pearson’s r = −0.61, *p* < 0.001). To discern whether *FTO*'s associations with neuroticism and well-being were independent, we adjusted for one trait while analyzing the other. Interestingly, the significance of the rs1421085 *FTO* genotype on neuroticism disappeared when adjusting for well-being, and likewise, the *FTO* genotype's significance on well-being disappeared when adjusting for neuroticism (Table 2; model c, B: 0.08, *p* = 0.11, and Table 3; model c, B: −0.29, *p* = 0.33; respectively). We further investigated the potential for neuroticism and well-being to either mediate or moderate the other’s effect in relation to the *FTO* genotype. Interaction terms were introduced in our regression models to assess moderation. These terms did not yield significant results, suggesting that neuroticism and well-being do not moderate the effect of the *FTO* genotype on each other (Tables S7 and S8). However, both cross-sectional and lagged longitudinal mediation analyses uncovered a significant indirect effect of rs1421085 on reduced well-being, which was mediated by higher neuroticism, in the models adjusted for age, sex, BMI, alcohol use, and childhood adversities (Table 4; cross-sectional, model b, Boot: −0.587, 95% CI [−1.040, −0.149]; longitudinal, model c, Boot: −0.433, 95% CI [−0.791, −0.069]). The adjusted lagged longitudinal mediation analysis of rs1421085 on well-being, with neuroticism as the mediator, is also illustrated as a path diagram (Fig. S4). Although there was also a significant effect when examining the impact of rs1421085 on neuroticism through well-being in the cross-sectional adjusted mediation analysis, no such effect was observed in the lagged longitudinal analyses (Table S9).

**Table 4:** Indirect effect of the *FTO* rs1421085 genotype^‡^ on well-being (PART wave III) through neuroticism measured in PART wave III^*^ or wave II^**^

N	Indirect Effect (Boot 95% CI)^a^	Sig	N	Indirect Effect (Boot 95% CI)^b^	Sig
2056	−0.418 (−0.870, 0.033)*^a^	No	1819	−0.588 (−1.034, −0.143)*^b^	Yes
2062	−0.250 (−0.625, 0.133)**^a^	No	1779	−0.433 (−0.786, −0.079)**^c^	Yes

Boot CI: Bootstrap Confidence Interval, Sig: Statistical significance.

^‡^T-allele carriers (CT or TT; coded 1) compared to CC homozygotes (CC; coded 0).

^*^Cross-sectional mediation analysis.

^**^Lagged longitudinal mediation analysis.

^a^Crude model.

^b^Adjusted for age, sex, BMI (PART wave III), AUDIT-10 (PART wave III), childhood adversities.

^c^Adjusted for age, sex, BMI (PART wave II and III), AUDIT-10 (PART wave II and III), childhood adversities.

### Exploring the indirect role of BMI in FTO’s association with well-being

Next, since BMI has been causally linked to well-being^48,49^ and since there was a modest but significant negative correlation between BMI and well-being in our replication cohort (Pearson’s r = −0.04, *p* < 0.001), we also examined the indirect effect of rs1421085 on well-being through BMI. Both cross-sectional and lagged longitudinal mediation analyses revealed a significant indirect effect of rs1421085 on enhanced well-being, which was mediated by lower BMI, in the models adjusted for age, sex, neuroticism, alcohol use, and childhood adversities (Table 5; cross-sectional, model b, Boot: 0.057, 95% CI [0.009, 0.127]; longitudinal, model c, Boot: 0.030, 95% CI [0.0004, 0.084]). The adjusted lagged longitudinal mediation analysis of rs1421085 on well-being, with BMI as the mediator, is also illustrated as a path diagram (Fig. S5). There was no correlation between neuroticism and BMI in the replication cohort (Pearson’s r = −0.002, *p* = 0.93), and no mediating effect was observed for the impact of rs1421085 on neuroticism through BMI in cross-sectional and longitudinal analyses (Table S10). Taken together, the mediation results highlight a complex role of the *FTO* gene in affecting well-being, illustrating that while the rs1421085 T-allele may be associated with reduced well-being through elevated neuroticism, it simultaneously appears to be linked to improved well-being via a reduction in BMI.

**Table 5:** Indirect effect of the *FTO* rs1421085 genotype^‡^ on well-being (PART wave III) through BMI measured in PART wave III^*^ or wave II^**^

N	Indirect Effect (Boot 95% CI)	Sig	N	Indirect Effect (Boot 95% CI)	Sig
2075	0.028 (−0.004, 0.085)*^a^	No	1819	0.058 (0.009, 0.133)*^b^	Yes
2081	0.019 (−0.007, 0.067)**^a^	No	1767	0.031 (0.0006, 0.083)**^c^	Yes

Boot CI: Bootstrap Confidence Interval, Sig: Statistical significance.

^‡^T-allele carriers (CT or TT; coded 1) compared to CC homozygotes (CC; coded 0).

^*^Cross-sectional mediation analysis.

^**^Lagged longitudinal mediation analysis.

^a^Crude model.

^b^Adjusted for age, sex, neuroticism (PART wave III), AUDIT-10 (PART wave III), childhood adversities.

^c^Adjusted for age, sex, neuroticism (PART wave II and III), AUDIT-10 (PART wave II and III), childhood adversities.

### Exploring the indirect role of neuroticism in FTO’s association with alcohol use

Direct associations between the *FTO* variant rs1421085 and alcohol use were not observed in the replication cohort (Table S4). However, given the association between rs1421085 and neuroticism (Table 2), and between neuroticism and alcohol use suggested by previous causal studies^50^, we hypothesized that neuroticism could mediate an indirect association between rs1421085 and alcohol consumption. Both cross-sectional and lagged-longitudinal adjusted mediation analyses uncovered significant indirect associations between rs1421085 and problematic alcohol use (AUDIT-P), that were mediated by higher neuroticism (Table 6, AUDIT-P; cross-sectional, model b, Boot: 0.042, 95% CI [0.011, 0.080]; longitudinal, model c, Boot: 0.019, 95% CI [0.0002, 0.043]). The adjusted lagged longitudinal mediation analysis of rs1421085 on AUDIT-P, with neuroticism as the mediator, is also illustrated as a path diagram (Fig. S6). Both crude and adjusted cross-sectional, but not longitudinal, analyses showed indirect associations between rs1421085 and AUDIT-10 via neuroticism (Table 6, AUDIT-10; cross-sectional, model a, Boot: 0.030, 95% CI [0.001, 0.071]; model b, Boot: 0.043, 95% CI [0.009, 0.088]). Interestingly, however, this mediating role of neuroticism did not extend to the AUDIT-C subscale, which does not capture problematic alcohol use (Table 6). We also examined indirect effects for rs1421085 on AUDIT scales through BMI but found no significances in the adjusted or lagged models (Table S11). In summary, while direct links between *FTO* and alcohol intake were not found in the replication cohort, our findings suggest again an indirect pathway, where higher neuroticism acts as a mediator between the *FTO* gene and problematic alcohol use.

**Table 6:** Indirect effect of the *FTO* rs1421085 genotype^‡^ on AUDIT scales (PART wave III) through neuroticism measured in PART wave III^*^ or wave II^**^

AUDIT scale	N	Indirect Effect (Boot 95% CI)	Sig	N	Indirect Effect (Boot 95% CI)	Sig
AUDIT-10	1983	0.030 (0.001, 0.071)*^a^	Yes	1890	0.043 (0.009, 0.087)*^b^	Yes
	1992	0.019 (−0.019, 0.060)**^a^	No	1853	0.001 (−0.012, 0.018)**^c^	No
AUDIT-C	1993	−0.003 (−0.017, 0.007)*^a^	No	1900	0.001 (−0.014, 0.017)*^b^	No
	2002	−0.001 (−0.012, 0.006)**^a^	No	1863	−0.017 (−0.039, 0.0003)**^c^	No
AUDIT-P	2003	0.034 (0.002, 0.073)*^a^	Yes	1910	0.042 (0.011, 0.078)*^b^	Yes
	2012	0.020 (−0.023, 0.064)**^a^	No	1872	0.019 (0.0006, 0.042)**^c^	Yes

AUDIT-10: Full AUDIT scale (10 items), AUDIT-C: Alcohol consumption items, AUDIT-P: Alcohol problem items, Boot CI: Bootstrap Confidence Interval, Sig: Statistical significance.

^‡^T-allele carriers (CT or TT; coded 1) compared to CC homozygotes (CC; coded 0).

^*^Cross-sectional mediation analysis.

^**^Lagged longitudinal mediation analysis.

^a^Crude model.

^b^Adjusted for age, sex, BMI (PART wave III), childhood adversities.

^c^Adjusted for age, sex, BMI (PART wave II and III), AUDIT-10 (PART wave II), childhood adversities.

## Discussion

Our study extends the well-documented role of the *FTO* gene in metabolic traits to include psychiatric traits, suggesting a broader functional role for *FTO* which could have implications for understanding the genetic basis of psychiatric disorders. Specifically, building upon the well-documented association between *FTO* gene variations and addiction phenotypes observed in the recent GWAS literature^3–8^, including FTO’s involvement in dopaminergic signaling^11^, our study probed for potential associations with a broader spectrum of psychiatric traits by means of a phenome-wide association study (PheWAS). The PheWAS for *FTO* uncovered 224–242 traits across 17 domains that survived the multiple-correction testing criteria. The most pronounced signals resided within the metabolic domain, mirroring the findings from prior GWAS on obesity and related phenotypes^1,2^. Within the psychiatric domain, the PheWAS revealed significant psychiatric traits that belonged to four main categories, including (i) substance use, (ii) chronotype/sleep, (iii) well-being, and (iv) neuroticism. Connections between *FTO* and the first two categories have already been established by previous GWAS literature. Specifically, besides the already described *FTO* associations with substance use traits^3–8^, previous GWAS studies have reported *FTO* associations with morningness and shorter sleep duration^51–53^. However, the PheWAS findings on well-being and neuroticism are less well-established, although some preliminary evidence exists. For instance, a recent Mendelian Randomization (MR) study suggested that the *FTO* gene contributes to the genetic variation of BMI that is causally linked to well-being^48^. In addition, a genetic study of a Korean traditional medicine system, which categorizes people into four constitutional types, reported an association between *FTO* and the (So-Eum) type that is characterized by high neuroticism^54^.

To corroborate the PheWAS findings, we aimed at replicating the associations of *FTO* with psychiatric traits using the PART study which had longitudinal data on BMI, alcohol use, well-being, and neuroticism (but not chronotype/sleep). To this end, we first set out to identify a proxy SNP with putative functionality that could be genotyped in this cohort. Among the eight *FTO* SNPs previously associated with substance use phenotypes in GWAS studies^3–8^, rs1421085 had the highest possible functional score on RegulomeDB suggesting its involvement in gene regulation and expression; a finding that was also supported by the Braineac and GTEx databases. Moreover, rs1421085 was the only variant that emerged as significant on the cross-ancestry level in three separate GWAS of alcohol use traits, including AUD^3^, problematic alcohol use^8^, and drinks per week^6^. MR findings also suggested that rs1421085 is the SNP with the largest contribution to the genetic variation in BMI that affects well-being^48^. Thus, despite the inherent limitations of single-variant analyses, the rs1421085 variant was chosen as a proxy SNP due to its robust functional evidence and the strong, consistent associations across different studies.

In our replication cohort, the significant association of rs1421085 with BMI was reaffirmed in both crude and adjusted linear regression models, supporting the established results on *FTO*'s influence on body mass^1,2^. When examining psychiatric traits, rs1421085 exhibited significant associations with neuroticism and well-being after adjusting for age, sex, BMI, alcohol use, and childhood adversities. Specifically, carriers of the T allele, previously implicated in a heightened risk for AUD^3^, showed higher levels of neuroticism and diminished well-being. Neuroticism constitutes a personality trait that is characterized by negative affectivity, including anxiety, anger, and emotional instability^55^. Therefore, it is noteworthy that FTO’s molecular target, i.e., RNA methylation, has been implicated in regulating stress response mechanisms in the brain^56^. Moreover, previous literature has consistently found neuroticism to be the personality trait that is most highly and negatively associated with different components of well-being, including happiness, life satisfaction, and quality of life^57^. An inverse significant correlation between neuroticism and well-being was also present in our replication cohort. Previous GWAS of neuroticism have found significant negative genetic correlations with subjective well-being, and MR analyses have supported the presence of bidirectional associations between neuroticism and well-being^58^. In the replication cohort, we investigated the potential for neuroticism and well-being to either mediate or moderate the other’s effect in relation to the *FTO* genotype. Through adjusted cross-sectional and longitudinal mediation analyses, we discerned a significant indirect effect of rs1421085 on well-being, with neuroticism acting as the mediator. Specifically, the T-allele of rs1421085 was associated with reduced well-being through elevated neuroticism; a relationship that aligns with the broader literature showing a negative correlation between these two traits. Thus, the T-allele may amplify these effects, suggesting that individuals with this allele experience higher neuroticism, which negatively impacts their well-being more than in individuals without this allele. Next, considering the MR-suggested causal connection between BMI and well-being implicating rs1421085^48^, we extended our mediation inquiries to encompass BMI. In line with the MR study^48^, our cross-sectional and lagged longitudinal mediation analyses uncovered a significant indirect influence of rs1421085 on subjective well-being, mediated by BMI. Specifically, the T-allele of rs1421085 was linked to enhanced well-being through its association with lower BMI. Taken together, our findings suggest that the T-allele's effect on well-being is multifaceted, possibly involving a complex interplay between additional genetic predispositions and psychological traits.

Moreover, contrary to our expectations, our replication cohort did not demonstrate a direct link between rs1421085 and alcohol consumption. Building on the association between rs1421085 and neuroticism observed in our study, and between neuroticism and alcohol use observed in previous studies^50^, we explored the concept of significant mediated effects in the absence of direct effects^59^. Our adjusted cross-sectional and lagged longitudinal analyses, revealed that neuroticism significantly mediated the relationship between rs1421085 and the AUDIT-P subscale. Interestingly, this mediation was not observed with the AUDIT-C subscale. The differential mediation effect of neuroticism on the relationship between rs1421085 and AUDIT-P versus AUDIT-C can be explained by the distinct nature of these two constructs. The AUDIT-P subscale measures problematic alcohol use, including symptoms of alcohol dependence and harmful consequences of drinking. This aspect of alcohol use is more closely linked to psychological distress and maladaptive coping strategies. In contrast, the AUDIT-C subscale measures general alcohol consumption, which encompasses a wider range of drinking behaviors, including social and recreational drinking that may not be directly driven by emotional distress. Thus, the influence of neuroticism may be more pronounced in problematic drinking behaviors, which are more likely to be a result of attempts to alleviate psychological distress. Specifically, given neuroticism's role in predisposing individuals to various psychopathologies, it has been suggested that maladaptive substance use may be an attempt to mitigate the heightened anxiety, dysphoria, and emotional instability associated with this personality trait^55^. In line with this assumption, recent MR studies have suggested a causal relationship between neuroticism and alcohol consumption^50^. Past research on mediation has suggested that additional psychological constructs, like well-being^30^ and coping strategies^60^, can also indirectly influence the association between genetic predispositions and drinking behaviors. Taken together, these findings underscore the potential of genetic factors to precipitate problematic alcohol use by influencing psychological mechanisms of negative reinforcement, which are central to the development of alcohol use disorders^61^.

In sum, our study is the first to establish a direct link between the *FTO* gene and neuroticism. Using *FTO*’s rs1421085 SNP as a proxy, our data also indicate that *FTO* can indirectly influence well-being and problematic alcohol use through heightened neuroticism. To provide an overall summary of the effects of *FTO*’s rs1421085 genotype on the psychiatric traits studied, we have compiled the key findings into Table S12. This table highlights the direct and indirect effects, the mediators involved, significant mediation results, and the moderation terms tested. These findings may have significant public health implications. Specifically, they highlight the role of *FTO* in psychiatric traits and align with the importance of considering neuroticism—a trait putatively susceptible to treatment^62^—in therapeutic strategies. However, further investigation is necessary to determine the therapeutic potential of targeting FTO, alongside additional longitudinal and translational studies to confirm and expand our understanding of these findings. In addition, while our study provides preliminary insights into the role of the rs1421085 variant in neuroticism, subjective well-being, and problematic alcohol use, we acknowledge the polygenic nature of these psychiatric traits. Future longitudinal mediation-based research may benefit from incorporating polygenic risk scores (PRS) and gene-wide analyses to capture the cumulative effect of multiple genetic variants. Such approaches could offer complementary insights and a more comprehensive understanding of the genetic architecture underlying these complex traits.

### Supplementary Information

Supplementary Information 1.

Supplementary Information 2.

## Data Availability

The data are not publicly available due to ethical and privacy restrictions. Data are available on reasonable request from the corresponding author.
